# Expediting approval for medical countermeasures to address high burden disease: an ethical justification to move beyond emergency use authorisation

**DOI:** 10.1136/bmjgh-2023-013480

**Published:** 2023-11-02

**Authors:** Mathew Mercuri, Kristy Hackett, Ross Upshur, Claudia Isabel Emerson

**Affiliations:** 1 Institute on Ethics & Policy for Innovation, McMaster University, Hamilton, Ontario, Canada; 2 Medicine, McMaster University, Hamilton, Ontario, Canada; 3 Dala Lana School of Public Health, University of Toronto, Toronto, Ontario, Canada; 4 Philosophy, McMaster University Faculty of Humanities, Hamilton, Ontario, Canada

**Keywords:** Health policy, Public Health, Infections, diseases, disorders, injuries

## Abstract

Addressing global health crises requires a receptive and expedient policy environment to minimise delays in making available potentially life-saving technologies. Over time, the policy environment has adapted to ensure that communities have expedited access to promising technologies, such as vaccines, that can mitigate morbidity and mortality. Emergency authorisations are one such policy mechanism. While these have been employed successfully for several diseases, such as influenza, Ebola and COVID-19, the policy mechanism is tied to contexts where key bodies have designated the disease an ‘emergency’, whereas no equivalent mechanism exists for those failing to acquire the designation (eg, malaria and tuberculosis). In this paper, we examine ethical issues associated with emergency authorisations. We argue that there is no moral difference between those diseases considered emergencies and many that fail to be designated as such with respect to impact on affected communities. Thus, tying access to an expedient policy mechanism for approval to an emergency designation is ethically unjustified—it should be based on considerations of risks and benefits, the disease burden and the values of the communities that carry those risks and not contingent on if the disease is designated an emergency. We suggest the need to further enhance the policy environment to ensure access to similar expedited approval programmes irrespective of if a disease is an emergency. Levelling the field for access to expedited approval programmes across diseases can help in moving towards achieving global health equity but is not a panacea.

**Summary Box**

- Emergency use authorisations have been used to get promising technology (eg, vaccines) into communities burdened by disease, but only diseases that are designated as an emergency (eg, Ebola, 2009 H1N1, COVID-19) can benefit from this policy mechanism.
- We argue that there is no ethical justification for why some diseases (eg, Ebola, 2009 H1N1) should have access to a policy mechanism, while other diseases (eg, malaria, tuberculosis) that may carry an equal or even greater burden do not.
- We argue that policy mechanisms for expediting approval should not be tied to designation of a disease as an emergency.
- The policy environment should evolve to ensure access to similar expedited approval programmes for high burden diseases, irrespective of if a disease is designated an emergency.

## Introduction

Addressing global health crises requires a receptive and expedient policy environment to minimise delays in making available potentially life-saving technologies. Over the past few decades, several countries have introduced programmes for expediting approval (eg, fast track, priority review, accelerated approval), each to address limitations in the policy environment at the time and provide flexibility in adapting to public need.^1 2^ Emergency approval mechanisms—for example, the Emergency Use Authorisation (EUA) in the USA and the WHO Emergency Use Listing (EUL)—are a more recent example of expansion in the policy environment and serve as important administrative tools for expediting access to technologies that act as countermeasures to public health crises. Emergency approvals offer more flexibility in the evidentiary requirements but with more conditions compared with other expedited approval programmes.^3^

To qualify for an EUA/EUL the technology must target a disease that is considered an ‘emergency’. In practice, that means technology for some diseases can benefit from an approval mechanism, whereas others cannot, which may be concerning if the latter carry an equal or even greater burden of morbidity and mortality. Is there an ethically significant difference between 2009 H1N1 influenza and malaria, such that one should have access to more tools in the policy armament? The stipulation that a disease be considered an ‘emergency’ to have access to some forms of expedited approval (where there is no equivalent for disease not receiving that designation) may result in a missed opportunity in getting technologies that may mitigate suffering much faster into communities that need them. It is also unfair.

In this paper we examine some of the ethical issues associated with emergency use policy mechanisms. First, we briefly describe EUA/EUL and their criteria for use. Next, we examine how an emergency is defined by those with the authority to set EUAs/EULs in motion. We then highlight some examples of where emergencies were designated and question why that applies in one case and not others, such that the former has access to a wider set of policy tools. Finally, we raise the idea of expanding our policy toolkit to include mechanisms equivalent to EUA/EUL that do not rely on the designation of an emergency. Our hope is that our examples can generate discussion on how to improve the policy environment to give more flexibility in responding to global health crises.

Our analysis has several caveats. First, we cannot cover all policies in all contexts. As such, we have focused on the EUA and EUL because they are widely studied in the literature and are highly influential in global health. Second, our intended scope is unproven therapeutic or preventative interventions that do not yet have sufficient evidence of efficacy and safety to receive full authorisation but are ‘promising’ insofar as a competent authority (eg, public health experts, qualified scientific committee) considers there to be a positive balance of benefit and risk in the context where they will be applied. Third, EUAs/EULs are a relatively newer policy mechanism, and there is limited evidence of their impact. Indeed, some have raised concerns about expansion of EUA/EUL type policies to non-pandemic diseases.^4^ Any proposed policy mechanism should guard against putting into use harmful or futile interventions. Expanding these frameworks to include ‘non-emergency’ diseases will need to be accompanied with conceptual and empirical work to ensure they are meeting the needs of the community. Fourth, any expansion of approval mechanisms will not alone eliminate disease burden, but must be considered as part of a larger public health and research policy environment.^5 6^ Finally, while our examples focus on infectious disease, we are not suggesting a policy environment be restricted to that—our concerns may equally apply to non-infectious diseases.^5^

## Emergency authorisation policy mechanisms: EUAs and EULs

The US first introduced the EUA in 2004 under section 564 of the Federal Food, Drug and Cosmetic Act to allow the Food and Drug Administration (FDA) to strengthen ‘the nation’s public health protections against chemical, biological, radiological, and nuclear (CBRN) threats including infectious diseases’ through the use of ‘medical countermeasures’ (MCMs) needed during public health emergencies.^7^ The EUA policy applied to products not previously approved or for wider spread off-label use of approved products. Prior to this policy (the 2002 Bioterrorism Act, a precursor to EUAs, notwithstanding), pre-approval was generally limited to research (eg, Investigational New Drug).^8^ EUAs have since been initiated to support countermeasures for Anthrax, H1N1 influenza, MERS-CoV, Ebola, Zika and more recently for COVID-19.^9^

An EUA can be granted only after the declaration of an emergency, one that impacts national security. After a declaration, the FDA may consider limited approval for products where (1) there is belief it may be effective as a countermeasure, (2) the known benefits outweigh the known potential risks and (3) an approved alternative does not exist (or is not available).^10 11^ An EUA is temporary. It can be terminated at the end of a prespecified term or at the discretion of the FDA, for example, if a suitable alternative becomes available and is approved. It is also terminated when the declaration of an emergency is rescinded.^12^

The WHO EUL is described as a ‘risk-based procedure for assessing and listing unlicensed vaccines, therapeutics and in vitro diagnostics with the ultimate aim of expediting the availability of these products to people affected by a public health emergency’.^13^ The EUL is meant to advise member states about technology use where the ‘community may be more willing to tolerate less certainty about the efficacy and safety of products, given the morbidity and/or mortality of the disease and the shortfall of treatment and/or prevention options’. (This quote is from the WHO news archive on 7 July 2015, under ‘WHO Emergency Use Assessment and Listing Procedure (EUAL) for candidate vaccines for use in the context of a public health emergency’ and has since been removed. See Smith *et al*^14^) It was first developed after the Ebola outbreak in West Africa in 2014 under the Emergency Use Assessment and Listing, and later updated to the EUL in 2020.^14^

To be eligible for an EUL, a technology must meet four criteria: (1) the targeted disease is considered ‘serious or immediately life threatening, has the potential of causing an outbreak, epidemic or pandemic’, (2) existing products have been ineffective—that is, they have failed to eradicate disease or prevent outbreaks, (3) the manufacture of the technology complies with best practice standards, (4) the applicant will complete development and seek product licensure. Products having received an EUL include vaccines for Ebola, poliomyelitis and COVID-19.

## What makes for an emergency?

Although processes exist for designating an emergency, the decision is not value-free. The warrant for consideration of an EUA is a declaration of an emergency by the Secretary of the Department of Homeland Security, Department of Defence and/or Health and Human Services and can be made for an emerging infectious disease or pandemic where there is concern it may threaten national security.^7^ As such decisions are part of a political process, they are made on a case-by-case basis and there is room for judgement about disease impact. What is not clear is if the decision is primarily a technical consideration of risk (eg, a threshold for mortality is met), or if it requires other considerations (and if so, what are they?). If several factors are considered, it is not clear how they are weighted in making a decision. The consideration of national security also requires judgement—for example, when does widespread infection become a national security threat? It is possible to have a public health crisis that does not reach the status of a national security threat and yet warrants the need for expedited authorisation of a technology (eg, was the HIV/AIDS crisis in the 1980s a threat to national security?). However, it is not surprising that national security is a consideration given that the impetus for the policies that underwrite the EUA (eg, 2002 Bioterrorism Act; 2004 Project BioShield Act) were the 9/11 attacks on the USA.

One criterion for an EUL is the existence of a ‘public health emergency’, that is, a disease that is ‘serious or immediately life threatening’ with ‘potential of causing an outbreak, epidemic or pandemic’.^13^ The technical threat of a pathogen can be determined by science. What is not clear is if a particular threshold of risk must be met to be considered ‘serious’. There exist technical definitions of ‘outbreak’, ‘epidemic’ and ‘pandemic’. The consideration of ‘potential’ for these is more ambiguous and requires speculation and forecasting. Experience (eg, COVID-19, Ebola, H1N1 influenza) suggests that our ability to forecast disease impact is limited. It is also not clear if the criteria accommodate endemic disease that is ‘serious or life threatening’ or is limited to only those with potential for outbreak, epidemic or pandemic. Technical definitions and clear thresholds on technical risks may be helpful in resolving some of these concerns. However, the language in the EUL description seems to privilege acute cases, as is implied by ‘outbreak, epidemic, or pandemic’. The protracted risk of endemic disease may be equal to that of an outbreak/epidemic/pandemic disease. From an ethical standpoint, it should not matter when the deaths happen—they should be treated equally, conditional on similar scientific opportunity.^6^

An emergency warranting consideration of an EUL might fall under the declaration of a Public Health Emergency of International Concern (PHEIC) as part of the International Health Regulations (IHR). The PHEIC is defined as ‘an extraordinary event which is determined… (i) to constitute a public health risk to other States through the international spread of disease and (ii) to potentially require coordinated international response’,^15^ particularly in situations that are ‘serious, sudden, unusual or unexpected’. The decision to declare a PHEIC is determined under the advice of members of the emergency committee, convened by the Director General of the WHO and consisting of international experts. There have been seven declarations under the PHEIC since its inception in 2005: H1N1 influenza (2009), poliomyelitis (2014), Ebola (2014), Zika (2016), Ebola (2018), COVID-19 (2020) and monkeypox (2022).^16^ Although PHEIC is an important administrative tool for mobilising international agreements of WHO member states, it has received criticism,^16 17^ and is rarely used in the context of endemic diseases (polio seems to be the exception) that can have a destabilising effect on some communities or may require international mobilisation equal or greater to that of infectious diseases with a designation. The concern is that without a designation of PHEIC, international agreements, including consideration of an EUL, may not be triggered.

How we define an emergency (and how we decide it has ended) raises several ethical concerns. What counts as an emergency relies on who makes the designation and that not deemed politically expedient may be overlooked. A commitment to equity^18^ entails that what is an emergency should not depend on who it affects or who can benefit from a designation. Colloquial use of ‘emergency’ might also imply an acute concern. That could limit consideration of diseases with protracted risk, for example, endemic diseases, such as malaria, tuberculosis and HIV/AIDS, as ‘emergencies’. Should calling something an emergency depend on if it affects people in a short time versus the same number over a longer period? What counts as an emergency is a relative concept. From a global perspective, it is desirable that a process for designating an emergency is sensitive to a diversity of experiences, such that the interpretation of emergency criteria (eg, in the IHR) does not perpetuate existing inequities or create new ones.

## Emergency versus ‘non-emergency’ diseases

Diseases designated as an emergency that have products that qualified for EUA/EUL include Anthrax, H1N1 influenza, MERS-CoV, Ebola, Zika and COVID-19 under the EUA, and Ebola, poliomyelitis and COVID-19 under the EUL. The impact of these diseases on morbidity and mortality, and geographical location/size of the population affected or at high risk varies dramatically. Contrast the 2009 H1N1 influenza pandemic (11–18% of the global population infected,^19^ 18 449 confirmed deaths by the WHO,^20^ considered an underestimation^21^), with MERS-CoV, (much lower incidence, <1000 confirmed deaths since 2012^22^) or with COVID-19 (widespread infection, >6 million confirmed deaths worldwide by end of 2022). In each scenario, it was determined that the community would be willing to ‘tolerate less certainty about the efficacy and safety’ in light of the morbidity and mortality (either anticipated or experienced) presented by the disease and given what other treatments were available. Notably, several WHO priority diseases (ie, Crimean-Congo haemorrhaging fever, Marburg virus disease, Lassa fever, Nipah and henipaviral diseases, Rift Valley fever),^23^ have not received designations of PHEIC and no technology for these diseases has achieved EUL, raising the questions of what makes something a priority (vs an emergency) and what that means insofar as getting promising technologies to burdened communities.

Technical risks alone do not appear to drive the designation of an emergency. Consider the technical risk for diseases not designated as emergencies. Neglected tropical diseases are a classification of several pathogens responsible for close to 2 billion infections each year^24^ and half a million deaths.^25^ Each year malaria infects >200 million people resulting in 600 thousand deaths.^24^ For tuberculosis it is 10 million infections and 1.4 million deaths.^24^ Almost 40 million people are living with HIV, and >600 thousand die each year from HIV-related illness.^26^ The global burden of each of these diseases outstrips that for many emergency diseases.

The ‘non-emergency’ diseases do differ from those designated as emergencies. Many have shifted to an endemic phase and communities most burdened have learnt to live with them. However, learning to live with a disease does not entail that its impact ought to be less concerning. These diseases are also not novel—we have a good understanding of disease dynamics and their impact on health outcomes. We could speculate that specific diseases were designated emergencies due to fear about what might happen.^27^ Why uncertainty should give reason for greater flexibility in how approvals are determined compared to those circumstances where we know how a disease is decimating communities is not clear to us. Geography may also play a role—the burden of the ‘non-emergency’ diseases is concentrated in low and middle-income countries and not in high-income countries where those making emergency designations under the programmes we discuss often reside. If we consider all people to have the same intrinsic value, an important ethical principle in global health, it should not matter who carries the burden and where they live.^28–30^ The FDA and WHO, as well as other institutions in high-income countries, play an outsized role in global health and its policy environment because of their economic and political position. What they approve can dictate what technology is available. Policy is of little help if it is only used when the interests of powerful nations are at stake. Indeed, the policy landscape for expediting approvals changed dramatically in the USA in response to the AIDS crisis in the 1980s.^2^ Would we see malaria suddenly designated as an emergency if it began to re-emerge in high-income countries?

Another reason for the discrepancy might be the existence of therapies/strategies to manage the disease. There are antimalarial medications and environmental controls for malaria, triple therapy for AIDS and antibiotics for tuberculosis, whereas vaccines/antiviral therapies did not exist (or were not approved) for H1N1 influenza, Ebola or coronaviruses (MERS-CoV and COVID-19). Despite these technologies existing, the former diseases continue to cause significant morbidity and mortality—clearly existing strategies/technologies are failing. One could argue that we do not require additional technology approvals but instead more attention to overcoming barriers to use of existing technologies. However, new technologies might circumvent those (so far, intractable) barriers, but could be significantly delayed due to the policy environment. Consider RTS,S/AS01, a leading malaria vaccine candidate. First created in 1987, phase 3 testing completed in 2014 showed benefit in reducing risk of infection.^31^ Subsequent pilot implementation studies also suggested efficacy,^32 33^ while exhibiting a ‘favourable safety profile’.^33^ The WHO recommended widespread use in October 2021,^34^ 7 years after phase 3 trials showed potential benefit and 34 years after initial development. Given the high burden of malaria in many communities, is it unreasonable to expect that a community suffering from malaria might ‘tolerate less certainty about the efficacy and safety’ of a novel technology if it showed promise in alleviating that burden? Was that promise only realised after the pilot implementation studies? It is possible that such delays are due to unique challenges for developing malaria vaccines or observed efficacy (relatively lower than vaccines receiving EUA/EUL for other diseases), but in contrast to what was achieved for vaccines among emergency designated diseases (H1N1, Ebola, COVID-19), one might wonder about the marginal contribution of the policy environment.

We argue that there is no moral difference between those diseases considered emergencies and many that fail to be designated as such with respect to human suffering, social and economic disruption and negative impacts on human flourishing. If we are serious about health equity, preventing future outbreaks and meeting the Sustainable Development Goals, we need to be more thoughtful about *why* we make such arbitrary distinctions and *why we do not* use currently available tools to address pressing health needs. Perhaps we need to end thinking about global/public health in terms of emergencies and health security and focus our attention on developing policies and treaties that avoid the inconsistencies and concerns we have raised so far.

## Building a level playing field in our policy landscape: challenges and potential solutions

Uptake of any technology may be harmful, but it may be ethical to authorise that technology for use if not using it results in even more harm. Indeed, one cannot determine that without adequate research, but the global community appears to have different standards for different diseases. That is, some diseases have access to policy mechanisms, in the form of EUA/EUL, that can mean earlier access to promising technologies by those in need, whereas other diseases that may carry an equal or even greater burden of morbidity and mortality do not. It may be appropriate to have differing standards, but should that not depend on the impact of the disease and what is known about the technology rather than on whether the disease is designated as an emergency? Similar arguments have been made in reference to the ethical allocation of public research funding, that is, funding should be proportional to burden (which includes both prevalence and severity,) conditional on the scientific opportunity.^6^

We can approach the problem we raised in several ways. First, we can lobby to have high burden diseases designated as an emergency, thereby opening the door for use of an emergency authorisation were a promising technology to come along. That approach does not solve the problem of how we determine an emergency and may still result in lost opportunities to initiate a policy mechanism to address an important global/public health issue. Second, we can make better use of the expedited approval programmes that are available, such as the FDA fast track, accelerated approval or the expanded access pathway.^4^ However, this may not be a satisfactory solution to the issues we raise here, as there would still be a policy mechanism available for some diseases and not for others due to a designation rather than the burden they present. Another approach is to develop a more general mechanism that would not require a designation of an emergency but will consider the willingness to take a risk on a promising technology relative to the disease’s impact on a community. Such a mechanism would allow for more consistency in our thinking on global and public health. As we have argued, an ethical justification is lacking for tethering an approval process exclusively to an emergency designation. We suggest that the warrant for authorisation should be contingent on the weight a community gives to the risks and benefits of using a promising technology relative to the burden of disease on that community.

## Limitations to expanding expedited approval mechanisms

Importantly, expedited approvals are not a panacea. Changes to the evidence standard, even under the condition that research will continue during the approval term, can risk patient exposure to an unsafe or ineffective technology.^35^ Furthermore, they risk public hesitancy if there is a perception that important steps are skipped. An acceptable level of rigour must be met to avoid harming patients and to ensure continued trust by those who stand to benefit from the technology (and carry the risks).^36 37^ Public engagement may be helpful in mitigating these concerns, but engagement can be difficult to undertake and brings with it several other ethical questions (eg, who should be included, how is that decided, how to weigh public concerns with those of scientists and decision-makers?). (Nevertheless, recent experience with COVID-19 suggests it is possible to conduct robust public engagement even in the most challenging of circumstances.^38^) Emergency authorisations (or their equivalent) can shape the research and development landscape with negative repercussions.^39^ There is ambiguity about what is meant by ‘innovation’ when it comes to unproven interventions that has consequences for policy.^40^ It is important that decision-makers have a consistent and defensible standard for designating a technology as showing promise. Finally, expedited approvals, in any form, will not alone achieve equity in global health—levelling the field for access to expedited approval programmes across diseases does not ensure that what is approved will be equitably distributed in the community.

## Conclusion

We advocate for a policy environment that is sensitive not only to the known risks and benefits of the technology (and the uncertainty around those estimates), but also to the burden of risk from the disease and the values of the communities that carry those risks. We currently have a valuable policy mechanism in emergency authorisations that has no equivalent for several high burden diseases. A fair system is one that weighs the risks and benefits of intervening with the risks and benefits of not, irrespective of who is being cared for and if the international community deems it an emergency. The threshold for action should be sensitive to the context.

## Data Availability

There are no data in this work.
